# Epicardial Connection as a Resource of Atrial Fibrillation Recurrence After Pulsed Field Ablation: A Case Report

**DOI:** 10.1002/ccr3.73282

**Published:** 2026-10-02

**Authors:** Kyong Hee Lee, Takeshi Kitamura, Shuichi Sahashi, Haruki Sugiyama, Chihiro Izumi, Kentaro Hayashi

**Affiliations:** ^1^ Department of Cardiology, Arrhythmia Center Ageo Central General Hospital Saitama Japan; ^2^ Department of Cardiology Tokai University School of Medicine Isehara Japan; ^3^ Department of Clinical Engineering, Arrhythmia Ageo Central General Hospital Saitama Japan

**Keywords:** atrial fibrillation, epicardial connection, FARA pulse, left common pulmonary vein, ligament of mashall, pulsed field ablation, recurrence of atrial fibrillation

## Abstract

This case highlights an uncommon but clinically important mechanism of atrial fibrillation recurrence after Pulsed field ablation. It emphasizes the importance of ablation along the ridge during the first session and also the need for careful evaluation of epicardial conduction, particularly in patients with a left common pulmonary vein.

## 1 Introduction

Pulsed field ablation (PFA) is a promising modality for atrial fibrillation (AF), selectively targeting myocardial tissue and sparing adjacent structures. Despite high acute success rates, long‐term durability and the mechanisms of recurrence remain unclear. Herein, we describe a rare case of AF recurrence due to an epicardial connection (EC) after PFA. This case highlights an uncommon but clinically relevant mechanism of AF recurrence following PFA.

## 2 Case History/Examination

A 55‐year‐old man presented with a two‐year history of palpitations. During symptomatic episodes, a 12‐lead electrocardiogram (ECG) revealed irregular narrow QRS tachycardia at 111 beats per minute with complete right bundle branch block morphology and left axis deviation (−21°). Transthoracic echocardiography demonstrated preserved left ventricular function and no structural abnormalities. Thyroid function tests were within normal limits. High‐resolution, contrast‐enhanced computed tomography revealed the left common pulmonary vein (PV) anatomy.

## 3 Differential Diagnosis, Investigations, and Treatment

The patient underwent ablation for symptomatic paroxysmal AF under general anesthesia (propofol and fentanyl), using supraglottic airway ventilation (i‐gel). Following rocuronium bromide administration, PFA was performed using the FARAPULSE system (Boston Scientific, Menlo Park, CA). Twenty‐two, ten, and eight applications were delivered to the left common PV, right superior, and right inferior PVs, respectively. To ensure adequate contact between the FARAPULSE catheter and the PV, fluoroscopy and intracardiac echocardiography (ViewFlex Xtra ICE, Abbott) were employed. The FARAPULSE application was set to 2.0 kV with biphasic mode and 4 pulse trains per application. Eight applications with basket configuration and 14 applications with flower configuration were performed to the left common PV. Six applications with basket configuration and 4 applications with flower configuration were performed to the right superior PV. Two applications with basket configuration and 6 applications with flower configuration were performed to the right inferior PV. Before application, the contact was checked by intracardiac echocardiography and fluoroscopy. Post‐ablation electro‐anatomical mapping (EnSite NavX, Abbott) confirmed acute PV isolation.

Three months later, AF recurrence was documented with a one‐week external event monitor, and a repeat procedure was performed. Under general anesthesia, an esophageal temperature probe was employed. Venous access was obtained from the right internal jugular and right femoral veins, and a dual‐decapolar catheter was positioned in the coronary sinus. Electro‐anatomical mapping was performed using the OctaRay mapping catheter and the CARTO3 v8 system (Biosense Webster). This patient provided written informed consent before undergoing the procedure.

At the start of the second procedure, premature atrial contractions (PACs) triggering AF were observed spontaneously. Initial 3D mapping of the premature atrial contractions was performed. The earliest activation site was localized beneath the left common PV ostium on the posterior wall, but it was located slightly apart from the first PV isolation line. The PACs were transiently suppressed by radiofrequency applications on this area with 35 W target AI 400 (QDOT Micro catheter, Biosense Webster). However, it failed to permanently eliminate it (Figure 1). Subsequent 3D electro‐anatomical voltage mapping during right atrial pacing demonstrated reconnection of the left PV, with ripple mapping identifying the anterior carina as the earliest site of reconduction (Figure 2). When 3D electro‐anatomical mapping was reconstructed with pacing from the left common PV, the site with the earliest local activation time (LAT) corresponded to the previously identified, non‐eliminated PACs origin. To further delineate the conduction pathway, pacing was performed from four distinct sites within the left common PV (posterior bottom, posterior roof, anterior ostium, between anterior ostium and posterior roof), and conduction times were measured from pacing sites to the distal coronary sinus electrode. The longest conduction times were observed from the posterior bottom site (140 ms), whereas the shortest was from the anterior ostium (30 ms). These findings suggested that reconnection was more consistent with an EC rather than an endocardial gap (Figure 3), although the endocardial latency gap could not be definitively excluded. Based on these observations, we made an assumption that conduction occurred via ECs, which connected between anterior carina of left PV and the left common PV ostium on the posterior wall. Therefore, this presumed epicardial pathway was targeted for ablation using the QDOT Micro catheter with 35 W and a target AI of 500. Consequently, the isolation of the left PV was completed, and no further arrhythmias were inducible. The success of PV isolation at the anterior carina supports the presence of an EC as the mechanism of reconduction rather than endocardial latency gap.

**FIGURE 1:**
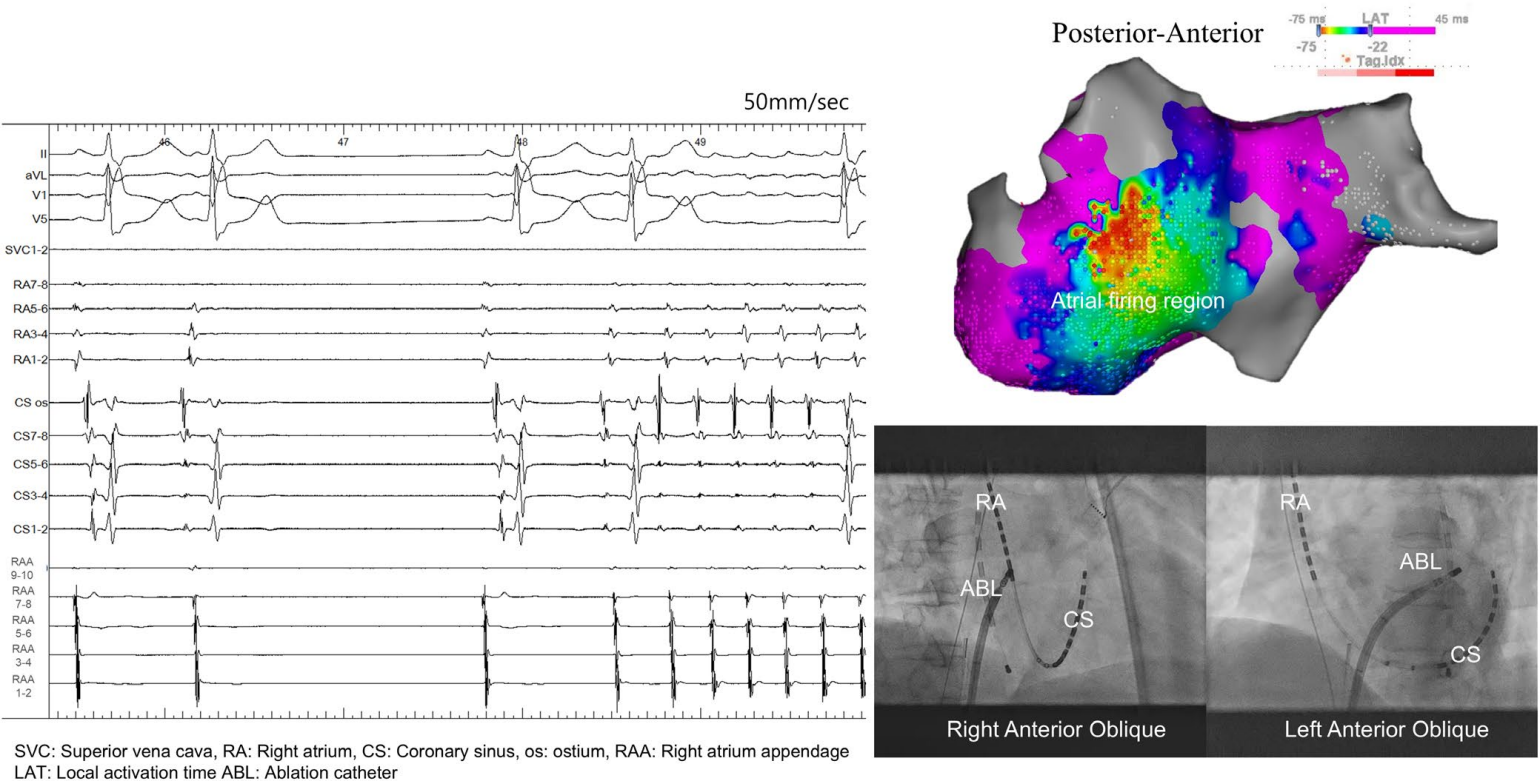
The firing atrial rhythm. Activation map of spontaneous premature atrial contractions triggering atrial fibrillation. The earliest activation site was beneath the left common pulmonary vein (PV) ostium on the posterior wall; however, it was located slightly apart from the first PV isolation line. Although the premature atrial contractions were transiently suppressed by radiofrequency applications in this area, they failed to permanently eliminate the former.

**FIGURE 2:**
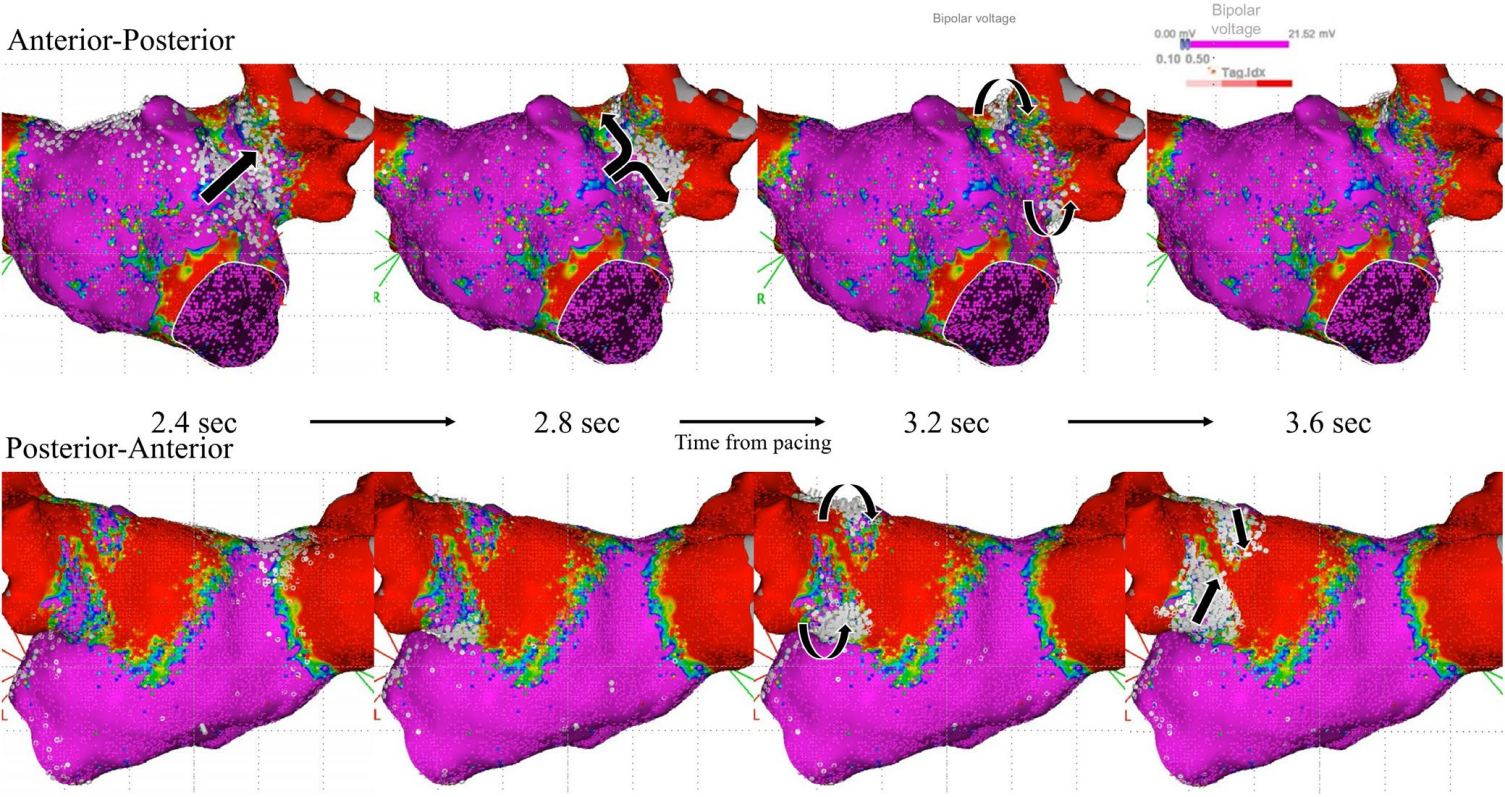
The three‐dimensional electro‐anatomical mapping. Electro‐anatomical voltage mapping during pacing from the right atrium revealed the reconnection of the left pulmonary vein, with the anterior carina as the earliest reconnected site on ripple mapping, followed by the roof and floor, and then the posterior wall.

**FIGURE 3:**
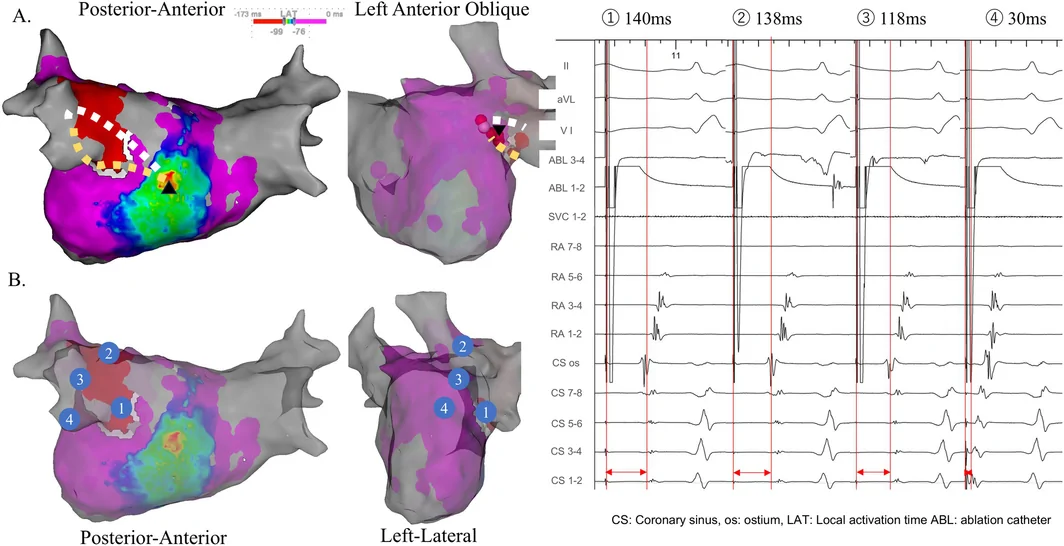
Reconstructed three‐dimensional electro‐anatomical mapping pacing on the reconnected region. (A) Electro‐anatomical mapping was reconstructed with pacing from the left common pulmonary vein (PV), the site with the earliest local activation time (LAT) corresponded to the previously identified, non‐eliminated premature atrial contractions (PACs) origin (▲). The yellow and white dotted lines indicate pathways predicted to represent epicardial conduction between the left anterior carina and the posterior PV ostium, which are presumed to involve the LOM or a left‐sided vein–atrium epicardial connection. Left PV isolation was completed by applying energy at the inferior portion of the anterior carina (▼). (B) To further delineate the conduction pathway, pacing was performed from four distinct sites within the left common PV (posterior bottom (①), posterior roof (②), anterior ostium (④), between anterior ostium and posterior roof (③)), and conduction times were measured from pacing sites to the distal coronary sinus electrode. The respective conduction times were measured as 140 ms, 138 ms, 118 ms, and 30 ms and the longest conduction times was observed from the posterior bottom site, whereas the shortest was from the anterior ostium.

## 4 Conclusion and Result

After procedure, the anti‐arrhythmic and beta‐antagonist drugs were not used, only one anticoagulant used for 6 months. During a year follow‐up period after the procedure, 24‐h Holter monitoring and 12‐lead electrocardiography were performed in the outpatient setting, revealing no recurrence of AF. The patient remained asymptomatic throughout the observation period. We plan to perform 24‐h Holter monitoring again annually thereafter.

We report a rare case of AF recurrence after PFA due to an EC. The durability of PFA ablation, especially in patients with a left common PV, remains unclear. Therefore, careful evaluation and consideration of epicardial reconnection should be performed, especially in patients with a left common PV, to achieve efficacy and durability.

## 5 Discussion

PFA is a novel ablation modality that uses irreversible electroporation to selectively ablate cardiomyocytes while minimizing collateral injury to surrounding anatomical structures such as the esophagus and phrenic nerve. Reported acute PV isolation rates are high, and early safety profiles are favorable when compared to thermal energy sources [1, 2, 3, 4]. Despite its promise, the long‐term validity and durability of PFA procedures remain uncertain relative to conventional treatment [5].

In this case, high‐resolution mapping revealed that AF recurrence originated from the left PV and propagated to the left posterior inferior wall of the left atrium via an EC. Importantly, the atrial premature contraction could not be suppressed by focal ablation of the posterior wall trigger site alone but required ablation at the anterior carina site, where the epicardial pathway exited the left posterior atrium. This suggests that while PFA achieved acute PV isolation, EC could bypass otherwise durable endocardial lesion sets.

EC can explain residual PV potentials and preserved atrial capture despite apparent entrance/exit block after circumferential PVI, with an incidence of approximately 11%–14% among patients with AF and a greater number of ECs in patients with advanced AF and enlarged left atria. High‐density mapping studies have demonstrated that most ECs are located at the PV carina and, sometimes, extend far distal to the antral line, typically conducting from right PVs to the high right atrium or His region and from left PVs to the coronary sinus, indicating epicardial muscular bundles linking PV sleeves to atrial myocardium [6, 7].

Anatomically, ECs arise from subepicardial bundles such as the septo‐pulmonary bundle, the intercaval bundle, interatrial groove muscle bridge (included Bachmann's bundle), coronary sinus musculature, and marshall bundle, which can maintain conduction across endocardial ablation lines until lesions become fully transmural [6, 8, 9, 10] Right‐sided ECs primarily link the right PV to the RA. They arise from intercaval and terminal‐crest‐related fibers on the RA posterior wall and course epicardially toward the right PV carina. These bundles attach to the posterior aspect of the right PV antrum. The interatrial groove is a fold or furrow formed where the anterior walls of the right and left atria meet. It appears as a groove extending obliquely from the superior wall of the right atrium (near the SVC) to the anterosuperior wall of the left atrium. The most significant muscular bridge located in this area is Bachmann's bundle, a subepicardial muscle bundle that originates from the upper portion of the right atrium's terminal crest, crosses anterior to the SVC, and continues into the anterosuperior wall of the left atrium. Left‐sided ECs are dominated by the ligament of Marshall (LOM) and its myocardial component, the Marshall bundle. From the coronary sinus, the vein of Marshall ascends within the LOM along the epicardial surface between the left atrial appendage (LAA) and the left superior pulmonary vein. Marshall bundle fibers then connect the CS, LA posterior and lateral wall, LAA ridge, and the left PV antrum, creating a subepicardial conduction network. This architecture explains ECs between LSPV and LA as well as complex loops involving the LAA–left superior PV ridge and posterior LA. Additional left‐sided vein–vein EC and vein–atrium EC have been described, particularly around superior left PVs or common trunks, where epicardial fibers interlink PVs or connect PV ostial regions to the LA roof and posterior wall [11, 12, 13]. In this case, we could not identify which epicardial connection (EC) was involved because neither an electrode catheter nor ethanol infusion was used; thus, the EC was inferred only from the post‐pacing interval. The EC between the left anterior carina and the posterior PV ostium was presumed to involve the LOM or a left‐sided vein–atrium EC rather than an endocardial connection (Figure 3). However, high‐resolution mapping and the successful ablation site suggested that these ECs may have contributed to AF recurrence after PFA.

In a study by Kueffer et al., the left common PV had the lowest durability in PFA cases [14]. In patients with a left common PV, a variant of left PV anatomy, the LOM is anatomically closer, increasing the potential for ECs capable of bypassing standard endocardial ablation lines. Consequently, lower recurrence rates have been reported in AF patients with left common PV treated using radiofrequency energy [15, 16]. However, lower durability has been reported for left common PV ablation using the cryoballoon [17]. Per these findings, unlike point‐by‐point radiofrequency ablation, FARAPULSE‐guided PFA, as a one‐shot device like the cryoballoon, may be more vulnerable to anatomical variants such as a left common PV.

Nevertheless, this case report has several limitations that should be acknowledged. First, being the report of a single case, the findings are not generalizable to all patients with a left common PV. Second, during treatment, we assumed the presence of an EC via the LOM or a left‐sided vein–atrium EC and performed ablation based on that assumption; however, we did not place an electrode catheter to confirm this assumption. Therefore, although we demonstrated the existence of an EC, we could not definitively prove the detailed pathway.

## Author Contributions

**Takeshi Kitamura:** data curation, investigation, validation, conceptualization, methodology. **Kyong Hee Lee:** writing – original draft, writing – review and editing, visualization. **Haruki Sugiyama:** data curation, investigation, validation. **Shuichi Sahashi:** data curation, investigation, validation. **Kentaro Hayashi:** conceptualization, writing – review and editing, supervision, investigation, methodology. **Chihiro Izumi:** visualization, data curation.

## Funding

The authors have nothing to report.

## Consent

Written informed consent was obtained from the patient for the publication of this case report.

## Conflicts of Interest

The authors declare no conflicts of interest.

## Data Availability

The data that support the findings of this study are available from the corresponding author upon reasonable request.
